# PROTOCOL: Risk factors for femicide

**DOI:** 10.1002/cl2.1123

**Published:** 2020-10-28

**Authors:** Monica Caicedo‐Roa, Tiago Da Veiga Pereira, Ricardo Carlos Cordeiro

**Affiliations:** ^1^ Department of Collective Health, Faculty of Medicinal Sciences State University of Campinas (UNICAMP) Campinas São Paulo Brazil; ^2^ International Research Center Hospital Alemão Oswaldo Cruz Health Technology Assessment Unit São Paulo São Paulo Brazil; ^3^ Applied Health Research Centre, Li Ka Shing Knowledge Institute St. Michaelʼs Hospital Toronto Ontario Canada; ^4^ Department of Health Sciences University of Leicester Leicester United Kingdom

## 1 BACKGROUND

### 1.1 Description of the condition

Femicide is the killing of women, girls and baby girls because of their gender. Femicide is the violent death of women based on gender, whether it occurs within the family, a domestic partnership, or any other interpersonal relationship; in the community, by any person, or when it is perpetrated or tolerated by the state or its agents, by action or omission (OAS 2008). Femicide constitutes a violation of women's rights and can be understood as, the ultimate form of violence against women, which ends in the killing of a woman or women affected. The term femicide was used since 1976 by the sociologist Diana Russell, with the objective of emphasizing differences in the characteristics of the women and men homicides (Russell, 2011). Femicide became frequently used in academic research, epidemiology, public health, politics, social science, laws, policy making even several conceptual theories that have been expanded through observational epidemiological studies.

The concept of femicide could be too confusing and too broad because of the gender component. International researchers have made efforts to investigate about femicide concept (Sanz‐Barbero et al., 2016). Aware of the importance of a clear and operational definition of femicide for data analysis and monitoring systems, an extended definition of femicide taking into account the cultural aspects and the possibility of the women act as aggressor and commit femicide. Femicide could be perpetrated by intimate partners, family members, and in rare occasions the perpetrators can be women either lesbian partners or kin (Weil et al., 2018; WHO, 2012). Then, the most recent definition of femicide is: *The killing of a woman because some man or men, although occasionally also some women who accept men*ʼ*s values, has or have sentenced her to death adducing whatever reasons, motives or causes, but nonetheless actually and ultimately because he or they believe she has defied (the words they often use are “offended” or “insulted”) patriarchal order (in their words “honourable” societies) beyond what her judge (often but not always the same person who kills her) is prepared to tolerate without retaliating in that way* (Grzyb et al., 2018; Iranzo, 2015).

Femicide is used in general for conceptualized forms of discrimination and violence against women when it is present an unequal power distribution, sometimes with government complicity and as a result of a cultural constructions. Femicide was translated to Spanish as *feminicide* and it is used in Latin American countries for the characterization of the cases and in laws. In this review, we will use the terms femicide and feminicide as synonyms.

Killing of women based on this gender is a global issue. There are cases in all countries in the world, they are tolerated, accepted, justified and could remain unpunished (ACUNS, 2014; Sarmiento et al., 2014). The killing of a woman by her partner is often the culmination of long‐term violence and can be prevented (UNODC, 2019). Women are killed with firearms, knives, or brute force, depending on the circumstances of the incident, the type of perpetrator, and other contextual factors, such as the presence of firearms in the home (Geneva Declaration Secretariat, 2015). Estimates accounts for 137 women killed in the world daily by a family member (UNODC, 2019). Even though men are the principal victims of lethal violence, women continue to bear the heaviest burden as a result of gender stereotypes and inequality. Across the world, in rich and poor countries, in developed and developing regions, a total of 50,000 women per year are killed by their current and former partners, fathers, brothers, mothers, sisters, and other family members because of their role and status as women (UNODC, 2019).

Several types of gender‐related killings of women have been identified: intimate femicide, non‐intimate femicide, child femicide, family femicide, femicide because of association/connection, unorganized systematic sexual femicide, organized systematic sexual femicide, femicide because of prostitution or stigmatized occupations, femicide because of trafficking, femicide because of smuggling, transphobic femicide, lesbophobic femicide, racist femicide and femicide because of female genital mutilation (Sarmiento, 2014). Also can be included femicide for accusations of sorcery/witchcraft, dowry deaths and female selective abortions. All of them have a gender component.

### 1.2 Description of the exposition

Gender‐related killings are the extreme manifestation of existing forms of violence against women. Such killings are not isolated incidents that arise suddenly and unexpectedly, but represent the ultimate act of violence which is experienced in a continuum of violence (United Nations, 2012). Femicide is the result of multiple, increasing and continuous manifestations of violence, which are rooted in the historical unequal power relations between men and women and in the systemic gender‐based discrimination, supported by pseudo‐social values, cultural patterns and practices (ACUNS, 2014).

Several risk factors have been identified related to a woman being victim of femicide: prior domestic violence, gun access, estrangement, threats to kill and threats with a weapon, nonfatal strangulation, and stepchild in the home if a female victim. Other risks included stalking, forced sex, and abuse during pregnancy (Campbell et al., 2003).

Some factors have been identifiable in men for abusing his partner too. The world report of violence has collected them and organized in categories as follows (Krug et al., 2002).

- **Individual factors:** young age, heavy drinking, depression, personality disorders, low academic achievement, low income, witnessing, or experiencing violence as a child, being abused during childhood, absent, or rejecting father.
- **Relationship factors:** marital conflict, marital instability, male dominance in the family, economic stress, poor family functioning, isolation of the woman from her family.
- **Community factors:** weak community sanctions against domestic violence, poverty, unemployed, low social capital, delinquent peer association.
- **Societal factors:** traditional/rigid gender norms/roles, social norms supportive of violence, sense of ownership over women.

### 1.3 How the exposition might work

The analysis of violence phenomena must recognize the influence of cultural factors constructed around the roles and behavior of men and women and the diminished power of women explained by lack of access to resources. The pursuit of a single explanatory factor is inadequate. Approximations as intersectionality (Sosa, 2017) and the ecological frameworks/model have been applied to conceptualized violence against women integrating individual, situational/relationship, exosystem/community, and macrosystem/societal factors (Heise, 1998; Krug et al., 2002) or theoretical approaches (Corradi et al., 2016).

There are several risk factors for femicide as mentioned above, the major one intimate partner homicide is prior domestic violence (Campbell et al., 2007). Evidence indicates that the majority of gender motivated killings of women are perpetrated by intimate partners or close family members (UNODC, 2011). Women are 9 times as likely to be killed by an intimate partner (husband, boyfriend, same‐sex partner, or ex) than by a stranger (Campbell et al., 2007). Globally, as many as 38% of all murders of women are committed by intimate partners (WHO 2013). Home is the most likely place for a woman to become a victim of homicide (UNODC, 2011).

Women are often emotionally involved and economically dependent on those who victimize them contributing to the perpetuation and acceptance of violence (Ellsberg et al., 2000; Krug et al., 2002). In general, various types of abuse coexist, for example, sexual, physical, economical, moral, patrimonial, and psychological abuse. Manifestations of violence increase with time and become more severe causing the death of the woman. Not all the femicides occur in this context, but most of them do.

The median time that women spend in a violent relationship is around 5–10 years depending on the womanʼs age. Justifications to continue in a violent relationship include fear of retribution, lack of alternative means of economic support, concern for children, emotional dependence, a lack of support from family or friends, an abiding hope that the abusive man will change, and the stigmatization associated with being unmarried (Ellsberg et al., 2000; Krug et al., 2002).

A womanʼs response to abuse is often limited by the options available to her, having into account the lack of positive response of society. Traditional societies defend menʼs rights of physically punishing their wives based on cultural and religious justifications (Ellsberg et al., 2000; Krug et al., 2002).

Violence against women have several and deep overall consequences. Abusive partner relationships have deep impact on womenʼs health (physical, sexual, reproductive, physiological, behavioral, and fatal health consequences (United Nations, 2015)). Fatal health consequences could be: AIDS‐related mortality, maternal mortality, femicide and forced suicide (Krug et al., 2002). The resulting damage also extends its impacts on the health of children. In fact, children may suffer a range of behavioral and emotional disturbances, included psychological, social, physical, and academic consequences e.g., post‐traumatic stress, attachment difficulties, weight and appetite changes, and drops in school grades (Alisic et al., 2015). The experience of violence episodes during childhood can be associated with perpetrating or experiencing violence later in life (Renner 2006).

### 1.4 Why it is important to do this review

Femicide is a research priority because of fatal consequences and protection of the women human rights. Femicide is still remarkably prevalent in many cultures and societies.

What constitutes a “femicide or feminicide” is not entirely clear, and how the concept has been defined within observational research is not universally standardized. This systematic review will explore how femicide has been conceptualized and evaluated in currently available case‐control and cohort studies.

Furthermore, we will systematically identify factors associated with the risk of femicide.

A better understanding of its risk factors can help the development of interventions as well as novel preventive strategies to mitigate this problem.

## 2 OBJECTIVES

Our main objective is to systematically identify factors associated with the risk of femicide. Besides, we will investigate how femicide has been defined by researchers.

### 2.1 Review question

How has femicide been defined in the epidemiological case‐control and cohort studies?

Which are the main risk factors for femicide?

**Table:** 

P	Population	Women (any age)
E	Exposition	Any risk factor for femicide (to be identified)
C	Comparator	Nonexposure to risk factors
O	Outcome	Definition of femicide used in available studies
		Social and demographic characteristics of the victims and aggressors
		Risk factors to be a victim of femicide
		Risk factors for a person to commit a femicide
		Mechanism of femicide‐related death
		Location of the femicide
		Relationship between victims and perpetrators
		Motivation/justification of the perpetrator
T	Study types	Observational studies (case‐control and cohort studies).

## 3 METHODS

### 3.1 Criteria for considering studies for this review

#### 3.1.1 Types of studies

We will include both prospective and retrospective cohort studies as well as case‐control studies, irrespective of sample size, year of publication or publication status (i.e., both published and nonpublished studies will be acceptable). Studies must be written in English, Spanish, or Portuguese.

##### Inclusion criteria

- Case‐control or cohort studies that investigated risk factors for feminicide
- Study that has a group of women with the homicide outcome and another one for comparison (population controls, women victims of mild/severe violence, women killed controls, women with attempted homicide, other)
- Studies that have a group of perpetrators of female homicide and a comparison group (population controls, men perpetrating another type of crime, other)
- Studies that report relative risk (RR) or odds ratio (OR) with the corresponding 95% confidence intervals (CIs) or that offer data to perform the calculation.

##### Exclusion criteria

- Studies without comparation group or case series.

#### 3.1.2 Types of participants

We will include studies that studied women victims of homicide. No restriction on age will be imposed.

#### 3.1.3 Types of expositions

We will include cases of femicide described as:

- Female homicide/femicide with firearm
- Female homicide/femicide with bladed weapon
- Female homicide/femicide with blunt object
- Female homicide/femicide by strangulation/asphyxiation
- Female homicide/femicide burn
- Female homicide/femicide by intoxication
- Other violent forms of female homicide/femicide

Any accidental deaths will not be considered for this systematic review.

#### 3.1.4 Types of outcome measures

It is important to notice that femicide is a relatively recent denomination of female homicide. We will include cases of female homicide. In cases of transsexual population, we will consider gender identity (e.g., if the biological sex of the person is male but gender identity is female, the person will be considered a woman). If it is not possible to establish the gender identity, we will include the particular case as gender identity undefined. We will extract 2 × 2 tables, RR or odds ratio‐related estimates—depending on the data availability in each study. When data are available as a point estimate (95% CI) derived from two or more multiple regression models, we will extract the estimates from the most complete model (full model).

##### Primary outcomes

- Definitions of femicide used in the primary case‐control and cohort studies.
- Associated factors to be a victim of femicide estimated by RRs or ORs or available data.
- Associated factors for a person to commit a femicide estimated by RRs or ORs or available data.

##### Secondary outcomes

Types of exposures to be included, observational studies must describe data on risk factors associated with femicide. Whenever possible, risk factors will be further categorized in victim‐related and perpetrator‐related factors. Examples of specific risk factors to be systematized:

- Age
- Race/ethnicity
- Immigrant status
- Educational level
- Employment
- Socioeconomic status
- Previous violence relationship
- Victim or witness of domestic violence during childhood (<14 years)
- Victim of rape or sexual violence in childhood (<14 years)
- Daughter of a mother battered by her partner
- History of depression
- History of mental illness
- Manic or psychotic symptoms
- Personality disorder
- Suicide attempt
- Firearm access
- Alcohol/drugs use
- Age difference of woman and partner
- Relationship between woman and partner
- Relationship duration
- Cohabitation
- Type of violence perpetrated by partner
- Type of violence perpetrated by women
- Increased frequency of physical violence over the past year
- Attempted femicide
- Previous of aggression of woman with a firearm or white arm
- Nonfatal attempt to strangle/hang woman by partner
- Rape or forced sex by partner
- History of murder of a relative
- Desire/attempt or separation by woman
- The woman had a new relationship
- Woman denounced violence to authorities
- Attempts to drop charges or going back on the decision to leave or report the aggressor to the police
- Woman with protective measure
- The woman had children
- Woman had nonbiological children with the abuser
- Woman had biological children with the abuser
- Physical aggression during pregnancy (from beginning to 12 months postpartum)
- Aggression to children by the partner
- Aggression of children with a firearm or white arm
- Violent behavior against people without inbreeding specification
- Violent behavior against people with inbreeding
- Violent behavior against people with no inbreeding/unknown ties
- Commit previous crimes
- Arrest history
- History of violent behaviors with previous partner
- Previous detention or history for domestic violence
- Jealous or controller behavior
- Cruel behaviors directed at the victim and lack of remorse
- Justification of violent behavior due to aggressorʼs own state (alcohol, drugs, stress) or victimʼs provocation
- Son of a mother raped by her partner

Additional risk factors not mentioned above be explicitly mentioned in the final version of the systematic review.

### 3.2 Search methods for identification of studies

We will search for all published and unpublished studies in the most common medical databases. We will include Google Scholar searcher to retrieve additional studies and relevant references from related systematic reviews.

#### 3.2.1 Electronic searches

We will use the following database from their earliest dates to March 2020. The search strategies are presented in the appendices:

1. Medline (Ovid platform) Appendix 1
  - Ovid MEDLINE(R) 1946 to 2020
  - MEDLINE(R) Epub Ahead of Print 2020
  - MEDLINE(R) Daily Update 2020

2. Embase (Ovid platform) Appendix 2
3. Scopus Appendix 3
4. Biblioteca Virtual da Saúde (BVS) Appendix 4
5. Web of Science Appendix 5
  - Principal Coleção do Web of Science
  - KCI‐Data base of Korean journals
  - Russian Science Citation Index
  - SciELO Citation Index
  - Derwent Innovations Index

6. Proquest Appendix 6
  - Applied Social Sciences Index & Abstracts (ASSIA)‎ (1987–nowadays)
  - Education Resources Information Center (ERIC‎) (1966–nowadays)
  - ProQuest Central‎ (1970–nowadays)
  - Sociological Abstracts‎ (1952–nowadays)
  - ProQuest Dissertations & Theses Global‎ ‎ (multidisciplinary–dissertations)

7. Cochrane Database of Systematic Reviews (web platform) Appendix 7
8. PsycINFO Appendix 8
  - APA PsycInfoAPA PsycInfo
  - APA PsycArticlesAPA PsycArticles
  - APA PsycBooksAPA PsycBooks

9. SocINDEX Appendix 9
  - SocINDEX with Full Text
  - CAPES FSTA Full Text Collection
  - CINAHL with Full Text

#### 3.2.2 Searching other resources

Publications from the World Health Organization, and the bibliographic references of included articles, as well as annals of scientific events on homicide in women, femicide and feminicide and the Opengrey literature databases (http://www.opengrey.eu) as complementary sources of information.

We will include the Google Scholar searcher (first 200 results) Appendix 10.

### 3.3 Data collection and analysis

#### 3.3.1 Selection of studies

We will download all titles and abstracts retrieved through the electronic search to EndNote online (Clarivate Analytics, 2015) and remove all duplicated references, we will transfer the data to the covidence platform (Covidence systematic review software) for the process of elaboration of the systematic review. Two reviewers will independently examine the referencesʼ reading titles and abstracts for identified primary studies. Disagreements will be resolved through discussion, or, if necessary, by consulting a third researcher. We will follow the PRISMA statements for the report selection process (Moher et al., 2009). We will use the Review Manager 5.4 for protocol and systematic review final text (The Cochrane Collaboration, 2014).

#### 3.3.2 Data extraction and management

We will independently extract study characteristics and outcome data from included studies using covidence; disagreements will be resolved through discussion, or if necessary, by consulting a third researcher. We will contact investigators by email to request further data on methods or results.

With data extracted by a standardized format (Table 1), we will elaborate tables of included, excluded (Table 2) and ongoing studies (Table 3). We will include studies based on type of studies (case‐control and cohort) and we will include all the relevant studies, regardless of the usability of the reported data. When several publications of the same study are found, we will choose the publication with more information and we will exclude the others, register them in the table of excluded studies, justified by the duplicated data presented.

**Table 1:** Table for data extraction

Supporting Information

**Table 2:** Characteristics of excluded studies

Reason for exclusion	Principal reason for exclusion
	

**Table 3:** Characteristics of ongoing studies

First author surname and year of publication	
Study name	
Methods	Type of study: Definition of femicide used:
Participants	Country: Inclusion criteria: Exclusion criteria:
Risk factors	Associated to the victim: Associated to the aggressor:
Outcomes	Primary outcomes: Secondary outcomes:
Starting date	
Contact information	
Notes	

We will compare the magnitude and direction of effects reported by studies using forest plot graphics and evaluating coherence of the data, trying to identify typographical errors in the studies reports.

#### 3.3.3 Assessment of risk of bias in included studies

For evaluation of the methodological quality of the studies, we will include the Newcastle Ottawa scale (Wells et al., 2019) for case‐control (Table 4) and cohort studies (Table 5). Process of assessment of risk bias will be in an independent way by the same authors who extracted data from the studies, and discrepancies resolved as mentioned above. We will provide a risk of bias table with the judgment and the justification of each item, and a summary graph of bias for each study and for all of them.

**Table 4:** Risk of bias table case control studies

Bias	Item	Authors judgment	Support for judgment
Selection	Is the case definition adequate?		
Representativeness of the cases			
Selection of Controls			
Definition of Controls			
Comparability	Comparability of cases and controls on the basis of the design or analysis		
Exposure	Ascertainment of exposure		
Same method of ascertainment for cases and controls			
Nonresponse rate			

**Table 5:** Risk of bias table cohort studies

Bias	Item	Authors judgment	Support for judgment
Selection	Representativeness of the exposed cohort		
Selection of the nonexposed cohort			
Ascertainment of exposure			
Demonstration that outcome of interest was not present at start of study			
Comparability	Comparability of cohorts on the basis of the design or analysis		
Exposure	Assessment of outcome		
Was follow‐up long enough for outcomes to occur			
Adequacy of follow up of cohorts			

We will mention quality of information in the results for the readers to be aware of possible bias derived from que quality of the information.

#### 3.3.4 Measures of treatment effect

##### Binary outcomes

Dichotomous data will be analyzed using numbers of events of each study, RRs or ORs, with 95% CIs.

##### Continuous outcomes

Continuous data will be analyzed with means (or mean changes) and standard deviations and will be summarized via standard mean differences.

In cases of missing standard deviations, we will recalculate them from the reported statistics provided in these studies (e.g. CIs, standard errors, *p* values).

If there are available data for two or more studies, we will combine available data for each outcome. We will combine risk ratios/odds ratios or standardized mean differences using a random‐effects model with the restricted maximum‐likelihood estimator of between—study variance. If there are more than 10 studies, contour‐enhanced plots will be constructed and statistical tests of funnel plot asymmetry will be performed (Eggerʼs test and Harbordʼs test). Heterogeneity will be assessed using both Cochranʼs *Q* test and the *I* ^2^ statistic. We will conduct sensitivity analyses and subgroups analyses in order to investigate possible sources of statistical heterogeneity. If the inconsistency is not explained by sensitive or subgroup analysis, and more than 10 studies are included in the meta‐analysis, a meta‐regression will be performed (Higgins et al., 2003).

#### 3.3.5 Unit of analysis issues

The unit of analysis is per dead woman.

#### 3.3.6 Dealing with missing data

We will attempt to contact study authors to obtain missing data. In cases of missing standard deviations, we will recalculate them from the reported statistics provided in these studies (e.g. CIs, standard errors, *p* values).

#### 3.3.7 Assessment of heterogeneity

Heterogeneity will be assessed using both Cochranʼs *Q* test and the *I* ^2^ statistic.

#### 3.3.8 Assessment of reporting biases

Review authors will aim to minimize the potential impact of reporting bias by ensuring the inclusion of the most important databases and resources to find relevant publications through the comprehensive search for eligible studies and by staying alert for duplication of data. If we include 10 or more studies in an analysis, we will use a funnel plot to explore publication bias and investigation of the relationship between effect size and study precision.

#### 3.3.9 Data synthesis

We will provide summary estimates of the strength of association between risk factors and femicide. Summary results will be obtained via a random‐effects models using the restricted maximum‐likelihood estimator of between‐study variance.

If there are more than 10 estimates per risk factor, countour‐enhanced plot will be constructed and statistical tests of funnel plot asymmetry will be performed (Eggerʼs test and Harbordʼs test). Heterogeneity will be assessed using both Cochranʼs *Q* test and the *I* ^2^ statistic. We will conduct sensitivity analyses and subgroups analyses in order to investigate possible sources of statistical heterogeneity. If the inconsistency is not explained by sensitive or subgroup analysis, and more than 10 studies are included in the meta‐analysis, a meta‐regression will be performed.

#### 3.3.10 Subgroup analysis and investigation of heterogeneity

We plan to perform a sensibility analysis based on the methodological quality of the studies and epidemiological design of the studies and analyses data for victims and aggressors.

#### 3.3.11 Sensitivity analysis

We will perform sensitivity analysis to determine the effects of the studies judged to be at “high” or “unclear” risk of bias.

#### 3.3.12 Overall quality of the body of evidence

We will prepare a summary of findings table using GRADEpro GDT 2014 software (GRADEpro 2015). This table will present the overall quality of the body of evidence according to GRADE criteria for the primary outcomes. These criteria include study limitations (i.e., risk of bias), consistency of effect, imprecision, indirectness, and publication bias (Balshem et al., 2011).

#### 3.3.13 Reaching conclusions

We will elaborate conclusions based only on findings from the synthesis (quantitative or narrative) of studies included in the review; we will avoid recommendations, but will recognize the implications of the findings for decision‐making, and we will talk about the remaining uncertainties.

## CONTRIBUTIONS OF AUTHORS

Monica Caicedo‐Roa: Conceived the review question, developed, completed and advised the protocol.

Tiago Da Vega Pereira: Developed, completed and advised the protocol.

Ricardo Carlos Cordeiro: Edited and advised the protocol.

## DECLARATIONS OF INTEREST

The authors declare no conflict of interests

## SOURCES OF SUPPORT

### INTERNAL SOURCES

- No sources of support provided

### EXTERNAL SOURCES

- Coordenação de Aperfeiçoamento de Pessoal de Nível Superior (Capes), Brazil.

## Supporting information

1 Table for data extraction

## APPENDIX 1 MEDLINE (OVID PLATFORM) SEARCH STRATEGY

1. exp domestic violence/
2. (domestic adj5 violence).ti,ab.
3. (family adj5 violence).ti,ab.
4. exp gender‐based violence/
5. (gender adj5 violence).ti,ab.
6. exp intimate partner violence/
7. (intimate adj5 partner adj5 violence).ti,ab.
8. (intimate adj5 partner adj5 abuse).ti,ab.
9. (dating adj5 violence).ti,ab.
10. (partner adj5 violence).ti,ab.
11. (partner adj5 abuse).ti,ab.
12. exp spouse abuse/
13. (spous$ adj5 abuse).ti,ab.
14. (wi#e adj5 abuse).ti,ab.
15. exp battered women/
16. (battered adj5 wom#n).ti,ab.
17. (abused adj5 wom#n).ti,ab.
18. (battered adj5 wi#e).ti,ab.
19. (wi#e adj5 beating).ti,ab.
20. exp women/
21. wom#n.ti,ab.
22. femal$.ti,ab.
23. girl$.ti,ab.
24. 1 or 2 or 3 or 4 or 5 or 6 or 7 or 8 or 9 or 10 or 11 or 12 or 13 or 14 or 15 or 16 or 17 or 18 or 19 or 20 or 21 or 22 or 23
25. exp homicide/
26. homicid$.ti,ab.
27. murde$.ti,ab.
28. killin$.ti,ab.
29. assassination.ti,ab.
30. 25 or 26 or 27 or 28 or 29
31. 24 and 30
32. femicid$.ti,ab.
33. feminicid$.ti,ab.
34. uxoricid$.ti,ab.
35. fratricide.ti,ab.
36. sororicide.ti,ab.
37. matricide.ti,ab.
38. parricide.ti,ab.
39. filicide.ti,ab.
40. (hono#r adj5 killin$).ti,ab.
41. 32 or 33 or 34 or 35 or 36 or 37 or 38 or 39 or 40
42. 31 or 41
43. exp observational study/
44. (observation$ adj5 stud$).ti,ab.
45. (non adj5 experimental adj5 stud$).ti,ab.
46. (nonexperimental adj5 stud$).ti,ab.
47. exp case‐control studies/
48. (case adj5 control adj5 stud$).ti,ab.
49. exp cohort analysis/
50. (cohort adj5 analysis).ti,ab.
51. (cohort adj5 stud$).ti,ab.
52. exp systematic review/
53. (systematic adj5 review).ti,ab.
54. 43 or 44 or 45 or 46 or 47 or 48 or 49 or 50 or 51 or 52 or 53
55. 42 and 54

## APPENDIX 2 EMBASE (OVID PLATFORM) SEARCH STRATEGY

1. ‘domestic violence’/exp
2. (domestic NEAR/5 violence):ti,ab,kw
3. (family NEAR/5 violence):ti,ab,kw
4. ‘gender‐based violence’/exp
5. (gender NEAR/5 violence):ti,ab,kw
6. ‘intimate partner violence’/exp
7. (intimate NEAR/5 partner NEAR/5 violence):ti,ab,kw
8. (intimate NEAR/5 partner NEAR/5 abuse):ti,ab,kw
9. (dating NEAR/5 violence):ti,ab,kw
10. (partner NEAR/5 violence):ti,ab,kw
11. (partner NEAR/5 abuse):ti,ab,kw
12. ʼspouse abuseʼ/exp
13. (spous* NEAR/5 abuse):ti,ab,kw
14. (wi?e NEAR/5 abuse):ti,ab,kw
15. ʼbattered womenʼ/exp
16. (battered NEAR/5 wom?n):ti,ab,kw
17. (abused NEAR/5 wom?n):ti,ab,kw
18. (battered NEAR/5 wi?e):ti,ab,kw
19. (wi?e NEAR/5 beating):ti,ab,kw
20. ʼwomenʼ/exp
21. wom?n:ti,ab,kw
22. femal*:ti,ab,kw
23. girl*:ti,ab,kw
24. #1 OR #2 OR #3 OR #4 OR #5 OR #6 OR #7 OR #8 OR #9 OR #10 OR #11 OR #12 OR 13 OR #14 OR #15 OR #16 OR #17 OR #18 OR #19 OR #20 OR #21 OR #20 OR #21 OR #22 OR #23
25. ʼhomicideʼ/exp
26. homicid*:ti,ab,kw
27. murde*:ti,ab,kw
28. killin*:ti,ab,kw
29. assassination:ti,ab,kw
30. #25 OR #26 OR #27 OR #28 OR #29
31. #24 AND #30
32. femicid*:ti,ab,kw
33. feminicid*:ti,ab,kw
34. uxoricid*:ti,ab,kw
35. fratricide:ti,ab,kw
36. sororicide:ti,ab,kw
37. matricide:ti,ab,kw
38. parricide:ti,ab,kw
39. filicide:ti,ab,kw
40. (hono?r NEAR/5 killin*):ti,ab,kw
41. #32 OR #33 OR #34 OR #35 OR #36 OR #37 OR #38 OR #39 OR #40
42. #31 OR #41
43. ʼobservational studyʼ/exp
44. (observation* NEAR/5 stud*):ti,ab,kw
45. (non NEAR/5 experimental NEAR/5 stud*):ti,ab,kw
46. (nonexperimental NEAR/5 stud*):ti,ab,kw
47. ʼcase‐control studiesʼ/exp
48. (case NEAR/5 control NEAR/5 stud*):ti,ab,kw
49. ʼcohort analysisʼ/exp
50. (cohort NEAR/5 analysis):ti,ab,kw
51. (cohort NEAR/5 stud*):ti,ab,kw
52. ʼsystematic reviewʼ/exp
53. (systematic NEAR/5 review):ti,ab,kw
54. #43 OR #44 OR #45 OR #46 OR #47 OR #48 OR #49 OR #50 OR #51 OR #52 OR #53
55. #42 AND #54

## APPENDIX 3 SCOPUS SEARCH STRATEGY

(((TITLE‐ABS‐KEY (“domestic violence”) OR TITLE‐ABS‐KEY (domestic W/5 violence) OR TITLE‐ABS‐KEY (family W/5 violence) OR TITLE‐ABS‐KEY (“gender‐based violence”) OR TITLE‐ABS‐KEY (gender W/5 violence) OR TITLE‐ABS‐KEY (“intimate partner violence”) OR TITLE‐ABS‐KEY ((intimate W/5 partner) W/5 violence) OR TITLE‐ABS‐KEY ((intimate W/5 partner) W/5 abuse) OR TITLE‐ABS‐KEY (dating W/5 violence) OR TITLE‐ABS‐KEY (partner W/5 violence) OR TITLE‐ABS‐KEY (partner W/5 abuse) OR TITLE‐ABS‐KEY ("spouse abuse") OR TITLE‐ABS‐KEY (spous* W/5 abuse) OR TITLE‐ABS‐KEY (wi?e W/5 abuse) OR TITLE‐ABS‐KEY ("battered women") OR TITLE‐ABS‐KEY (battered W/5 wom?n) OR TITLE‐ABS‐KEY (abused W/5 wom?n) OR TITLE‐ABS‐KEY (battered W/5 wi?e) OR TITLE‐ABS‐KEY (wi?e W/5 beating) OR TITLE‐ABS‐KEY ("women") OR TITLE‐ABS‐KEY (wom?n) OR TITLE‐ABS‐KEY (femal*) OR TITLE‐ABS‐KEY (girl*)) AND (TITLE‐ABS‐KEY ("homicide") OR TITLE‐ABS‐KEY (homicid*) OR TITLE‐ABS‐KEY (murde*) OR TITLE‐ABS‐KEY (killin*) OR TITLE‐ABS‐KEY (assassination))) OR (TITLE‐ABS‐KEY (femicid*) OR TITLE‐ABS‐KEY (feminicid*) OR TITLE‐ABS‐KEY (uxoricid*) OR TITLE‐ABS‐KEY (fratricide) OR TITLE‐ABS‐KEY (sororicide) OR TITLE‐ABS‐KEY (matricide) OR TITLE‐ABS‐KEY (parricide) OR TITLE‐ABS‐KEY (filicide) OR TITLE‐ABS‐KEY (hono?r W/5 killin*))) AND (TITLE‐ABS‐KEY ("observational study") OR TITLE‐ABS‐KEY (observation* W/5 stud*) OR TITLE‐ABS‐KEY ((non W/5 experimental) W/5 stud*) OR TITLE‐ABS‐KEY (nonexperimental W/5 stud*) OR TITLE‐ABS‐KEY ("case‐control studies") OR TITLE‐ABS‐KEY ((case W/5 control) W/5 stud*) OR TITLE‐ABS‐KEY ("cohort analysis") OR TITLE‐ABS‐KEY (cohort W/5 analysis) OR TITLE‐ABS‐KEY (cohort W/5 stud*) OR TITLE‐ABS‐KEY ("systematic review") OR TITLE‐ABS‐KEY (systematic W/5 review))

## APPENDIX 4 BIBLIOTECA VIRTUAL DA SAÚDE (BVS) SEARCH STRATEGY

tw:((tw:(tw:(((((mh:(domestic violence)) OR (tw:(domestic violence)) OR (tw:(family violence)) OR (mh:(violencia doméstica)) OR (tw:(violencia domestica)) OR (tw:(violencia familiar)) OR (mh:(violência doméstica)) OR (tw:(violência doméstica)) OR (tw:(violência na família))) OR ((mh:(intimate partner violence)) OR (tw:(intimate partner violence)) OR (tw:(intimate partner abuse)) OR (tw:(dating violence)) OR (tw:(partner violence)) OR (tw:(partner abuse)) OR (mh:(violencia de pareja)) OR (mh:(violência por parceiro íntimo)) OR (tw:(violência contra a parceira íntima)) OR (tw:(violência entre parceiros íntimos))) OR ((tw:(spous* abuse)) OR (tw:(wife abuse)) OR (tw:(battered women)) OR (tw:(battered woman)) OR (tw:(abused woman)) OR (tw:(battered wife)) OR (tw:(wife beating)) OR (mh:(maltrato conyugal)) OR (tw:(maltrato a la esposa)) OR (tw:(maltrato a la mujer)) OR (tw:(abuso de la pareja)) OR (tw:(síndrome de la esposa maltratada)) OR (mh:(maus‐tratos conjugais)) OR (tw:(maus‐tratos à parceira)) OR (tw:(maus‐tratos à companheira)) OR (tw:(maus‐tratos à esposa)) OR (tw:(síndrome da esposa espancada)))) AND ((mh:(homicide)) OR (mh:(homicidio)) OR (mh:(homicídio)) OR (tw:(homicídio)) OR (tw:(homicid*)) OR (tw:(murde*)) OR (tw:(killin*)) OR (tw:(assassination)) OR (tw:(asesinato)) OR (tw:(assassinato)))) OR ((tw:(femicid*)) OR (tw:(feminicid*)) OR (tw:(uxoricid*)) OR (tw:(fratricide)) OR (tw:(sororicide)) OR (tw:(matricide)) OR (tw:(parricide)) OR (tw:(filicide)) OR (tw:(hon* killing)))) AND (instance:"regional") AND (db:("LILACS" OR "INDEXPSI" OR "IBECS" OR "CUMED" OR "PAHOIRIS" OR "BDENF" OR "HANSENIASE" OR "LIS" OR "MedCarib" OR "BINACIS" OR "campusvirtualsp_brasil" OR "WHOLIS" OR "colecionaSUS" OR "PAHO" OR "tese" OR "BBO" OR "DESASTRES" OR "HISA" OR "HomeoIndex")))) AND (tw:(((mh:(observational study)) OR (tw:(observational study)) OR (tw:(observational study)) OR (tw:(observation* stud*)) OR (tw:(non experimental stud*)) OR (tw:(nonexperimental stud*)) OR (mh:(case‐control studies)) OR (tw:(case‐control studies)) OR (tw:(case control stud*)) OR (tw:(case control stud*)) OR (tw:(control stud*)) OR (mh:(cohort analysis)) OR (tw:(cohort analysis)) OR (tw:(cohort studies)) OR (tw:(cohort stud*)) OR (mh:(systematic review)) OR (tw:(systematic review))))))

## APPENDIX 5 WEB OF SCIENCE SEARCH STRATEGY

1. TS = “domestic violence”
2. TS = (domestic NEAR/5 violence)
3. TS = (family NEAR/5 violence)
4. TS = “gender‐based violence”
5. TS = (gender NEAR/5 violence)
6. TS = “intimate partner violence”
7. TS = ((intimate NEAR/5 partner) NEAR/5 violence)
8. TS = ((intimate NEAR/5 partner) NEAR/5 abuse)
9. TS = (dating NEAR/5 violence)
10. TS = (partner NEAR/5 violence)
11. TS = (partner NEAR/5 abuse)
12. TS = “spouse abuse”
13. TS = (spous* NEAR/5 abuse)
14. TS = (wi?e NEAR/5 abuse)
15. TS = “battered women”
16. TS = (battered NEAR/5 wom?n)
17. TS = (abused NEAR/5 wom?n)
18. TS = (battered NEAR/5 wi?e)
19. TS = (wi?e NEAR/5 beating)
20. TS = “women”
21. TS = wom?n
22. TS = femal*
23. TS = girl*
24. #1 OR #2 OR #3 OR #4 OR #5 OR #6 OR #7 OR #8 OR #9 OR #10 OR #11 OR #12 OR 13 OR #14 OR #15 OR #16 OR #17 OR #18 OR #19 OR #20 OR #21 OR #20 OR #21 OR #22 OR #23
25. TS = "homicide”
26. TS = homicid*
27. TS = murde*
28. TS = killin*
29. TS = assassination
30. #25 OR #26 OR #27 OR #28 OR #29
31. #24 AND #30
32. TS = femicid*
33. TS = feminicid*
34. TS = uxoricid*
35. TS = fratricide
36. TS = sororicide
37. TS = matricide
38. TS = parricide
39. TS = filicide
40. TS = (hono?r NEAR/5 killin*)
41. #32 OR #33 OR #34 OR #35 OR #36 OR #37 OR #38 OR #39 OR #40
42. #31 OR #41
43. TS = “observational study”
44. TS = (observation* NEAR/5 stud*)
45. TS = ((non NEAR/5 experimental) NEAR/5 stud*)
46. TS = (nonexperimental NEAR/5 stud*)
47. TS = “case‐control studies”
48. TS = ((case NEAR/5 control) NEAR/5 stud*)
49. TS = “cohort analysis”
50. TS = (cohort NEAR/5 analysis)
51. TS = (cohort NEAR/5 stud*)
52. TS = “systematic review"
53. TS = (systematic NEAR/5 review)
54. #43 OR #44 OR #45 OR #46 OR #47 OR #48 OR #49 OR #50 OR #51 OR #52 OR #53
55. #42 AND #54

## APPENDIX 6 PROQUEST SEARCH STRATEGY

((ti,ab(femicid*) OR ti,ab(feminicid*) OR ti,ab(uxoricid*) OR ti,ab(fratricide) OR ti,ab(sororicide) OR ti,ab(matricide) OR ti,ab(parricide) OR ti,ab(filicide) OR ti,ab(hono?r NEAR/5 killin*)) OR ((MJMESH.EXACT("domestic violence") OR ti,ab(domestic NEAR/5 violence) OR ti,ab(family NEAR/5 violence) OR MJMESH.EXACT("gender‐based violence") OR ti,ab(gender NEAR/5 violence) OR MJMESH.EXACT("intimate partner violence") OR ti,ab(intimate NEAR/5 partner NEAR/5 violence) OR ti,ab(intimate NEAR/5 partner NEAR/5 abuse) OR ti,ab(dating NEAR/5 violence) OR ti,ab(partner NEAR/5 violence) OR ti,ab(partner NEAR/5 abuse) OR MJMESH.EXACT("spouse abuse") OR ti,ab(spous* NEAR/5 abuse) OR ti,ab(wi?e NEAR/5 abuse) OR MJMESH.EXACT("battered women") OR ti,ab(battered NEAR/5 wom?n) OR ti,ab(abused NEAR/5 wom?n) OR ti,ab(battered NEAR/5 wi?e) OR ti,ab(wi?e NEAR/5 beating) OR MJMESH.EXACT("women") OR ti,ab(wom?n) OR ti,ab(femal*) OR ti,ab(girl*)) AND (MJMESH.EXACT("homicide") OR ti,ab(homicid*) OR ti,ab(murde*) OR ti,ab(killin*) OR ti,ab(assassination)))) AND (MJMESH.EXACT("observational study") OR ti,ab(observation* NEAR/5 stud*) OR ti,ab(non NEAR/5 experimental NEAR/5 stud*) OR ti,ab(nonexperimental NEAR/5 stud*) OR MJMESH.EXACT("case‐control studies") OR ti,ab(case NEAR/5 control NEAR/5 stud*) OR MJMESH.EXACT("cohort analysis") OR ti,ab(cohort NEAR/5 analysis) OR ti,ab(cohort NEAR/5 stud*) OR MJMESH.EXACT("systematic review") OR ti,ab(systematic NEAR/5 review))

## APPENDIX 7 COCHRANE DATABASE OF SYSTEMATIC REVIEWS (WEB PLATFORM) SEARCH STRATEGY

1. #1 MeSH descriptor: [Domestic Violence] explode all trees
2. #2 domestic violence:ti,ab,kw (Word variations have been searched)
3. #3 family violence:ti,ab,kw (Word variations have been searched)
4. #4 gender violence:ti,ab,kw (Word variations have been searched)
5. #5 MeSH descriptor: [Intimate Partner Violence] explode all trees
6. #6 intimate partner violence:ti,ab,kw (Word variations have been searched)
7. #7 intimate partner abuse:ti,ab,kw (Word variations have been searched)
8. #8 dating violence:ti,ab,kw (Word variations have been searched)
9. #9 partner violence:ti,ab,kw (Word variations have been searched)
10. #10 partner abuse:ti,ab,kw (Word variations have been searched)
11. #11 MeSH descriptor: [Spouse Abuse] explode all trees
12. #12 spous* abuse:ti,ab,kw (Word variations have been searched)
13. #13 wi?e abuse:ti,ab,kw (Word variations have been searched)
14. #14 MeSH descriptor: [Battered Women] explode all trees
15. #15 battered wom?n:ti,ab,kw (Word variations have been searched)
16. #16 abused wom?n:ti,ab,kw (Word variations have been searched)
17. #17 battered wi?e:ti,ab,kw (Word variations have been searched)
18. #18 wi?e beating:ti,ab,kw (Word variations have been searched)
19. #19 #1 or #2 or #3 or #4 or #5 or #6 or #7 or #8 or #9 or #10 or #11 or #12 or #13 or #14 or #15 or #16 or #17 or #18
20. #20 MeSH descriptor: [Homicide] explode all trees
21. #21 homicid*:ti,ab,kw (Word variations have been searched)
22. #22 murde*:ti,ab,kw (Word variations have been searched)
23. #23 killin*:ti,ab,kw (Word variations have been searched)
24. #24 #20 or #21 or #22 or #23
25. #25 #19 and #24
26. #26 femicid*:ti,ab,kw (Word variations have been searched)
27. #27 hono?r killin* (Word variations have been searched)
28. #28 filicide:ti,ab,kw (Word variations have been searched)
29. #29 #26 or #27 or #28
30. #30 #25 or #29

## APPENDIX 8 PSYCINFO SEARCH STRATEGY

((((((((Any Field: (family NEAR/5 violence))))) OR ((((MeSH: (gender‐based violence))))) OR ((((Any Field: (gender NEAR/5 violence))))) OR ((((MeSH: (intimate partner violence))))) OR ((((Any Field: (intimate NEAR/5 partner NEAR/5 violence))))) OR ((((Any Field: (intimate NEAR/5 partner NEAR/5 abuse))))) OR ((((Any Field: (dating NEAR/5 violence))))) OR ((((Any Field: (partner NEAR/5 violence))))) OR ((((Any Field: (partner NEAR/5 abuse))))) OR ((((MeSH: (spouse abuse))))) OR ((((Any Field: (spous* NEAR/5 abuse))))) OR ((((Any Field: (wi?e NEAR/5 abuse))))) OR ((((MeSH: (battered women))))) OR ((((Any Field: (battered NEAR/5 wom?n))))) OR ((((Any Field: (abused NEAR/5 wom?n))))) OR ((((Any Field: (battered NEAR/5 wi?e))))) OR ((((Any Field: (wi?e NEAR/5 beating))))) OR ((((MeSH: (women))))) OR ((((Any Field: (wom?n))))) OR ((((Any Field: (femal*))))) OR ((((Any Field: (girl*)))))) OR (((((MeSH: (domestic violence))))) OR ((((Any Field: (domestic NEAR/5 violence))))))) AND ((((MeSH: (homicide)))) OR (((Any Field: (homicid*)))) OR (((Any Field: (murde*)))) OR (((Any Field: (killin*)))) OR (((Any Field: (assassination)))))) OR (((Any Field: (femicid*))) OR ((Any Field: (feminicid*))) OR ((Any Field: (uxoricid*))) OR ((Any Field: (fratricide))) OR ((Any Field: (sororicide))) OR ((Any Field: (matricide))) OR ((Any Field: (parricide))) OR ((Any Field: (filicide))) OR ((Any Field: (hono?r NEAR/5 killin*))))) AND ((MeSH: (observational study)) OR (Any Field: (observation* NEAR/5 stud*)) OR (Any Field: (non NEAR/5 experimental NEAR/5 stud*)) OR (Any Field: (nonexperimental NEAR/5 stud*)) OR (MeSH: (case‐control studies)) OR (Any Field: (case NEAR/5 control NEAR/5 stud*)) OR (MeSH: (cohort analysis)) OR (Any Field: (cohort NEAR/5 analysis)) OR (Any Field: (cohort NEAR/5 stud*)) OR (MeSH: (systematic review)) OR (Any Field: (systematic NEAR/5 review)))

## APPENDIX 9 SOCINDEX WITH FULL TEXT SEARCH STRATEGY

1. DE "domestic violence"
2. AB domestic violence
3. AB family violence
4. DE "gender‐based violence"
5. AB gender violence
6. DE "intimate partner violence"
7. AB intimate partner violence
8. AB intimate partner abuse
9. AB dating violence
10. AB partner violence
11. AB partner abuse
12. DE "spouse abuse"
13. AB spous* abuse
14. AB wi?e abuse
15. DE "battered women"
16. AB battered wom?n
17. AB abused wom?n
18. AB battered wi?e
19. AB wi?e beating
20. DE "women"
21. AB wom?n
22. AB femal*
23. AB girl*
24. S1 OR S2 OR S3 OR S4 OR S5 OR S6 OR S7 OR S8 OR S9 OR S10 OR S11 OR S12 OR S13 OR S14 OR S15 OR S16 OR S17 OR S18 OR S19 OR S20 OR S21 OR S22 OR S23
25. DE "homicide"
26. AB homicid*
27. AB murde*
28. AB killin*
29. AB assassination
30. S25 OR S26 OR S27 OR S28 OR S29
31. S24 AND S30
32. AB femicid*
33. AB feminicid*
34. AB uxoricid*
35. AB fratricide
36. AB sororicide
37. AB matricide
38. AB parricide
39. AB filicide
40. AB hono?r killin*
41. S32 OR S33 OR S34 OR S35 OR S36 OR S37 OR S38 OR S39 OR S40
42. S31 OR S41
43. DE "observational study"
44. AB observation* stud*
45. AB non experimental stud*
46. AB nonexperimental stud*
47. DE "case‐control studies"
48. AB case control stud*
49. DE "cohort analysis"
50. AB cohort analysis
51. AB cohort stud*
52. DE "systematic review"
53. AB systematic review
54. S43 OR S44 OR S45 OR S46 OR S47 OR S48 OR S49 OR S50 OR S51 OR S52 OR S53
55. S41 AND S54

## APPENDIX 10 GOOGLE SCHOLAR (FIRST 100 RESULTS) SEARCH STRATEGY

1. ((femicidio) OR (feminicidio)) AND ((estudio* observacion*) OR (estudio* caso* control*) OR (analisi* cohort*) OR (estudio* cohort*) OR (revision sistematica))
2. ((femicide) OR (feminicide)) AND ((observation* stud*) OR (case control stud*) OR (cohort analysis) OR (cohort stud*) OR (systematic review))
